# Morphometrical malignancy grading is a valuable prognostic factor in invasive ductal breast cancer

**DOI:** 10.1038/sj.bjc.6600617

**Published:** 2002-11-12

**Authors:** P Kronqvist, T Kuopio, P Jalava, Y Collan

**Affiliations:** Department of Pathology, University of Turku, Turku, Finland; Department of Pathology, Jyväskylä Central Hospital, Jyväskylä, Finland

**Keywords:** breast cancer, prognosis, grading, morphometry

## Abstract

The aim of the present study is to augment the prognostic power of breast cancer grading by elaboration of quantitative histopathological methods. We focus on the recently introduced morphometrical grading system in which the three grading sub-features of the WHO grading system are evaluated with the help of computerised nuclear morphometry, and quantitative methods for assessing mitotic activity and tubular differentiation. The prognostic value of the morphometrical grading system is now confirmed in a material of 159 cases of invasive ductal breast cancer. In the current material the morphometrical grading system very efficiently predicted the prognosis of breast cancer by dividing the patients into favourable (grade I), intermediate (grade II), and unfavourable (grade III) outcome (*P*<0.0001). The morphometrical grading system was especially efficient in identifying patients with the most unfavourable outcome. In our material the morphometrical grade III was associated with a 5.4-fold risk of breast cancer death. In light of the present results, the morphometrical grading can be applied to clinical use as an aid in treatment decisions of patients with invasive ductal breast cancer.

*British Journal of Cancer* (2002) **87**, 1275–1280. doi:10.1038/sj.bjc.6600617 www.bjcancer.com

© 2002 Cancer Research UK

## 

Histological malignancy grading of invasive ductal breast cancer provides valuable information of the outcome of the patient (Parl and Dupont 1982, Davis *et al*, 1986, Contesso *et al*, 1987, 1989; Henson, 1988; Elston and Ellis, 1991; Simpson and Page, 1994; Roberti *et al*, 1997; Page *et al*, 1998). Standardised applications of the grading system have led to still higher accuracy in predicting the outcome of breast cancer (Theissig *et al*, 1990; Royal College of Pathologists Working Group, 1991; Elston and Ellis, 1991; Simpson and Page, 1994). According to the Nottingham Breast Cancer Study (Elston *et al*, 1982; Elston and Ellis, 1991) among grade III tumours half of the patients will die of the disease within 5 years from the diagnosis. Instead, practically every patient with a tumour of grade I is alive at 5 years of follow-up.

Since medical literature shows convincing evidence of the benefits of standardised methods in histological malignancy grading we have set out to intensify the grading of invasive ductal breast cancer by elaboration of quantitative morphometrical methods. For the first, we have introduced quantitative and computer-assisted histopathological measurement methods by which the extent of the three grading sub-features – nuclear pleomorphism, mitotic activity and tubular differentiation – can be expressed in numerical terms (Haapasalo *et al*, 1989; Kronqvist *et al*, 1995, 1997, 1999; Collan *et al*, 1996; Kuopio and Collan, 1996). Secondly, on basis of follow-up information of a patient material we have determined quantitative thresholds (Kronqvist *et al*, 1998a,b, 2000) for allocating each of the grading sub-features into scores 1, 2, and 3 (Patey and Scarff, 1928; Bloom and Richardson, 1957; Who, 1981). The purpose of the present study is to examine the prognostic value of the combined morphometrical grading criteria in invasive ductal breast cancer.

## MATERIALS AND METHODS

### Patient material

The study comprises a total of 159 patients diagnosed and treated with primary invasive ductal breast cancer at Turku University Hospital during the years 1988–1991 (Table 1

**Table 1:**
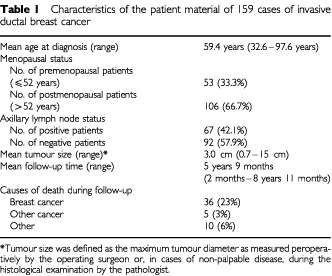
Characteristics of the patient material of 159 cases of invasive ductal breast cancer

). The material originates from a period when mammographic screening was already systematically practised in South Western Finland (Klemi *et al*, 1992, 1997). Complete follow-up histories and perioperative specimens were available from the primary tumours of each case. All patients were treated with radical or modified radical mastectomy and axillary evacuation. Preoperative adjuvant treatment was not administered, but 29% of the patients were treated with postoperative anti-oestrogen medication or cytostatic drugs. In Turku University Hospital district the indications for treatment with anti-oestrogens and cytostatic drugs changed at the turn of the decade. In the end of the 1980s, anti-oestrogens (tamoxifen) or cytostatic drugs were given to patients with a T4 stage disease. The same treatment was also given to patients detected with histologically verified metastasis in four or more axillary lymph nodes or in one or more apical lymph node. In the 1990's, in turn, adjuvant medication was given in all cases of histologically verified spread to axillary lymph nodes.

For detailed statistical analysis the material was stratified into prognostic subgroups. We used the age of 52 years at the time of diagnosis as a criteria for allocating the material into premenopausal and postmenopausal patient groups. This division was based on the reported average menopausal age in Finnish population (Saarikoski, 1986). Axillary lymph node status and tumour size were also used to divide the patients into prognostic subgroups. Axillary lymph node status was obtained from histological examination of the axillary fat pad. Tumour size as expressed in millimetres of the largest tumour diameter was obtained from peroperative measurement by the surgeon, or in cases of non-palpable tumours, from the histological section. We used tumour diameter 3 cm as the cut-point for allocating the patients into prognostic subgroups. In the present material tumour size 3 cm was shown to be the most efficient cut-point for prognostic stratification (*P*<0.0003).

The causes of death were based on autopsy reports, death certificates, and patient files. Breast cancer related survival rate was 71.3% as determined at 5 years of follow-up by excluding patients dead of causes other than breast cancer.

### Tissue processing

The tissue material prepared for morphometrical examinations was fixed in buffered formalin (pH 7.0), and embedded in paraffin. From a representative tissue block of each case one slide was prepared for morphometrical analyses. The tissue block was sectioned at 5 μm, and stained with haematoxylin and eosin. No pre-frozen tissue material was used in the measurements.

### Histological malignancy grading

Of all breast cancer cases in the present material two grades were available: the traditional subjective grade and the recently introduced morphometrical grade. The subjective grades (Bloom and Richardson, 1957; Who, 1981) were obtained from histology reports given in association with the original breast cancer diagnosis. The morphometrical grades were based on quantitative measurements of each of the grading sub-features and their numerical thresholds (Kronqvist *et al*, 1997, 1998b, 1999 2000).

#### Nuclear measurements for morphometrical grading

Nuclear morphometrical measurements were performed with the help of a digitising interactive video overlay drawing system run by the Prodit morphometry program (Prodit 3.1, Promis Inc., Almere, The Netherlands) (Table 2

**Table 2:**
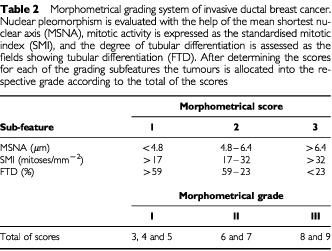
Morphometrical grading system of invasive ductal breast cancer. Nuclear pleomorphism is evaluated with the help of the mean shortest nuclear axis (MSNA), mitotic activity is expressed as the standardised mitotic index (SMI), and the degree of tubular differentiation is assessed as the fields showing tubular differentiation (FTD). After determining the scores for each of the grading subfeatures the tumours is allocated into the respective grade according to the total of the scores

). In addition to a standard light microscope the system included a personal computer (Compaq Deskpro 386/20e, Compaq Computer Corporation, Houston, TX, USA), a video camera (JVC TK-870U, JVC, Japan), a monitor screen (MultiSync 3D Color Monitor, NEC, Japan), and a digitiser board (PIP-512B video digitiser board, Matrox Electronic Systems, Dorval, Quebec, Canada). The nuclear profiles were measured by outlining their digitised images on the monitor screen with the computer mouse. Measurements were performed with ×40 objective magnification which when added to the ×10 video ocular and ×2 internal magnification resulted in an image magnification of ×2500 on the monitor screen. To ensure validity of results the morphometric instrument was carefully calibrated with a micrometre slide before each measurement session.

In each sample the measurement area was selected at the most cellular, usually peripheral part of the tumour which was considered to represent the invasive border of the tumour. These areas were marked with ink and were approximately 0.5 cm in diameter. Areas of necrosis, tissue artefacts, *in situ* carcinoma, and inflammation were avoided. In each microscopical field those malignant epithelial cell nuclei with clearly identified nuclear borders were selected. An average of 6–15 adjacent microscope fields were screened with a total of 50 consecutive tumour cell nuclei measured in each sample. The number of measured cells per sample was obtained from a pilot study comparing the value of different sampling rules in nuclear morphometry (Kronqvist *et al*, 1995). The optimal sample size can be theoretically confirmed when the desirable precision level and the probability of reaching that precision is defined (Collan *et al*, 1987). In the present study the measurement of one sample took approximately 10–15 minutes. After measurement of one sample was completed the program automatically calculated 11 morphometrical variables with their basic statistics. Five of the variables gave information about the size of the nucleus and the remaining six variables characterised the shape of the measured nuclear profiles. Among the morphometrical features describing nuclear size and shape and their variation, the thresholds determined for the shortest nuclear axis was associated with the highest statistical significances (Kronqvist *et al*, 1998a). The thresholds for the shortest nuclear axis (SNA) were therefore chosen optimal in stratifying cases on basis of their nuclear pleomorphism (Kronqvist *et al*, 1998) (Table 2).

#### Mitotic counts for morphometrical grading

Mitotic activities of each sample were assessed in the same measurement areas that were applied for nuclear morphometry. Mitoses were counted from 10 consecutive fields (objective ×40, field diameter 450 μm). Identification of a mitotic figure was based on the absence of the nuclear membrane and observation of at least one separate chromosome, usually seen as a small protuberance or a clear hairy projection at the outline of the mitotic figure. Special consideration was put on discrimination between mitotic figures and apoptotic bodies (Baak *et al*, 1989). In case of doubtful histological interpretation a mitosis was not registered. Two chromosome collections originating from one cell division were registered as one mitosis in distinction from the principle of Baak *et al* (1989).

The mitotic calculations were performed by two laboratory technicians specially trained for the task (Kuopio and Collan, 1996). In each microscopic field the mitotic count together with the observers' assessment on the area fraction of malignant epithelium was entered on a form. The resulting standardised mitotic activity (SMI) expressed the number of mitoses per square millimetre of malignant epithelium. Evaluation of one sample took in average 10–15 min. The grading thresholds for SMI were determined so that they optimally stratified cases into prognostic groups on the basis of their mitotic activity (Kronqvist *et al*, 1998b) (Table 2).

The special advantage of SMI is that it takes into account the amount of connective tissue within the tumour. This is especially significant in tumours with small cell density where the few mitoses of the malignant tissue are dispersed among the large amount of connective tissue. SMI was originally introduced by Haapasalo *et al* (1989) as volume corrected mitotic index (M/V index) but in a later paper on request of a referee the name was changed to standardised mitotic index (Collan *et al*, 1996). In the meantime, the name volume fraction corrected mitotic index (M/V_v_ index) was used in one article (Kujari *et al*, 1994). Also this change in terminology was demanded by a referee.

#### Evaluation of tubular differentiation for morphometrical grading

In assessing the degree of tubular differentiation in breast cancer samples special consideration was placed on histological identification of the malignant glands. A gland was defined as a tubular or alveolar structure comprising a group of cancer cells with a definite lumen at the centre. The basal location of the cancer cell nuclei in the neoplastic glandular epithelium, the so-called nuclear polarisation, was an additional criterion. Special caution was taken not to misinterpret artefactual clefts caused by tissue processing, adipocytes or central necrosis in a group of cancer cells for tubular structures. Luminal structures in cribriform intraductal or infiltrative epithelium as well as benign ducts within malignant tissue were excluded from the measurements.

Our quantitative method for determining the degree of tubular differentiation has proven accurate, reliable, and practical in comparison to other tested evaluation methods (Kronqvist *et al*, 1999). In this method tubular differentiation was assessed in the whole tumour area (Who, 1981; Elston and Ellis, 1991; Simpson and Page, 1994) by determining the presence or absence of malignant tubuli in each microscopical field (×25 magnification, field diameter 710 μm) (Kronqvist *et al*, 1999) (Table 2). If at least one unambiguous malignant tubular structure was identified the field was registered as positive. The number of positive fields in relation to the total number of fields in the sample was called the fraction of fields showing malignant tubuli (FTD). The evaluation of one sample took 15–20 min.

### Threshold analyses for morphometrical grading

For each of the above described morphometrically determined grading sub-features two numerical thresholds were determined in order to give quantitative scores to each of the grading sub-features. Threshold determinations were performed with the help of Kaplan–Meier analysis (Cutler and Ederer, 1958) and tested with the help of chi-squares and *P* values of log-rank tests so that the cut-points showing the best curve separation, i.e. the highest statistical significance, were considered to best distinguish patients with different outcome of disease (Kronqvist, 1998a; b). These cut-points represented the optimal grade limits and were chosen as thresholds for morphometrical grading (Table 2). Finally, we added together the scores based on morphometrical measurements and quantitative thresholds of the grading sub-features. The breast cancer cases of the present material were allocated to the morphometrical grades on the basis of the original guidelines of Bloom and Richardson (1957) (Table 2).

### Statistical analysis

Agreements between the traditional subjective, and morphometrical grading systems were analysed with the help of kappa coefficients (Landis and Koch, 1977; Kraemer, 1980; Wulff, 1981; Silcocks, 1992). Kappa coefficients were used to estimate the relation of the observed agreement to the expected random agreement of the grading systems. Grading efficiencies (GE) (Galen and Gambino, 1975; Collan, 1989; Collan and Pesonen, 1989; Collan *et al*, 1992, Kujari *et al*, 1994) were used to estimate consistencies with respect to the fraction of uniformly graded cases. GE's were determined from 3×3 tables that evaluate stratification of the cases into three grades by the subjective and morphometrical grading system.

The prognostic contributions of the two grading systems were demonstrated with the help of Kaplan–Meier analysis (Cutler and Ederer, 1958) by drawing survival curves on basis of breast cancer survival in the patient material (SAS System for Windows™ release 8.01, SAS Institute, Cary, NC, USA). *P*-values of log-rank test were used to test the statistical significances of differences between the curves. Univariate and multivariate analyses of Cox's regression were used to determine the prognostic efficiency of the morphometrical grading system. We compared the prognostic significances of the morphometrical grading system, and of the patients' age at diagnosis, tumour size, and axillary lymph node status. The associations of the different prognostic factors with breast cancer recurrence or death were quantified with ratios indicating relative risk (RR) and their 95% confidence intervals (95% CI) in univariate and multivariate analyses.

## RESULTS

In the present material the mean measurement value for MSNA was 5.8 μm (range 3.1–11.1 μm), for SMI 16.8 (range 0–83.9), and for FTD 31.3% (range 0–96.6%). On this basis 43% of the cases were allocated to morphometrical grade I, 40% to morphometrical grade II, and 16% to morphometrical grade III. The corresponding fractions for subjective grades I, II and III were 15, 54 and 31%, respectively.

In 45% of the cases the subjective and morphometrical grading systems resulted in identical grades. In 11 (7%) samples we observed a disagreement between two grades and in these cases there was a disagreement of the subjective grade III and the morphometrical grade I. GE and kappa were 0.711 and 0.170 (95% CI 0.060–0.279), respectively, indicating poor agreement between the traditional subjective, and morphometrical grading methods.

Figure 1

**Figure 1:**
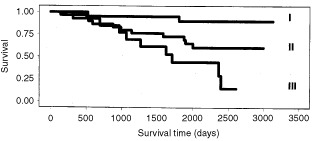
The morphometrical grading system separated the patients with tumours of grades I, II, and III with high statistical significance (*P*<0.0001).

shows the survival curves of the morphometrical grades. In the whole material the morphometrical grades were associated with a high prognostic significance (log rank test *P*<0.0001). In analysis of the patient subgroups the morphometrical grade showed prognostic significance among axillary lymph node positive (*P*<0.0005) cases, and separately among both premenopausal (*P*<0.0006) and postmenopausal patients (*P*<0.012).

**Figure 2:**
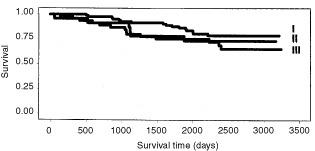
Survival curves for the subjective grades I, II, and III did not stratify the patients with different outcome of disease with statistical significance (*P*=0.275).

Instead, the subjective grading system did not separate with statistical significance patients with different outcome of disease in the whole material (Figure 2

) or in prognostic subgroups divided according to the patients' age at diagnosis, tumour size, and axillary lymph node status. In fact, in our material we observed the discrepancy of the cases representing subjective grade II showing a more favourable prognosis than those of subjective grade I.

Risk ratios (RR) of univariate analysis of Cox's regression are presented in Table 3

**Table 3:**
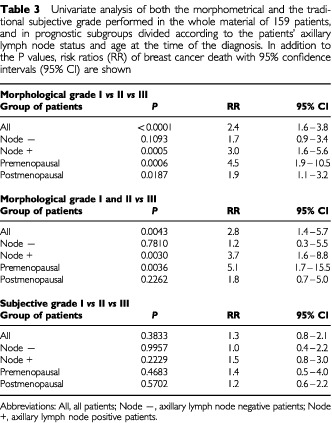
Univariate analysis of both the morphometrical and the traditional subjective grade performed in the whole material of 159 patients, and in prognostic subgroups divided according to the patients' axillary lymph node status and age at the time of the diagnosis. In addition to the P values, risk ratios (RR) of breast cancer death with 95% confidence intervals (95% CI) are shown

. The RR's of both morphometrical and subjective grading systems were produced by comparing the survival of patients associated with each grade. The RR's can be expected to give comparative estimates on the value of the different prognostic features. In analysis of the whole material, morphometrical grades I and II *vs* III were associated with a 2.8-fold risk of breast cancer death (*P*<0.005). Morphometrical grade reliably predicted the prognosis also among axillary lymph node positive and negative patients, and premenopausal patients (RR's ranging from 5.1 to 1.2). Instead, the traditional subjective grades did not predict patient outcome in our material with statistical significance.

The results of multivariate analysis (Table 4

**Table 4:**
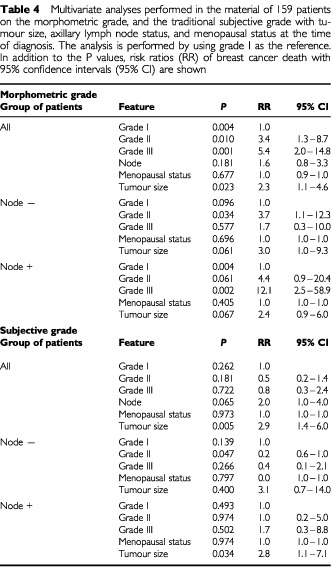
Multivariate analyses performed in the material of 159 patients on the morphometric grade, and the traditional subjective grade with tumour size, axillary lymph node status, and menopausal status at the time of diagnosis. The analysis is performed by using grade I as the reference. In addition to the *P* values, risk ratios (RR) of breast cancer death with 95% confidence intervals (95% CI) are shown

) show the relation between the evaluated prognostic features using grade I as the reference risk ratio. In our material the risk of breast cancer death increased 3.4-fold when the morphometrical grade rose from grade I to grade II (*P*<0.01). Between grades I and III the risk of breast cancer death increased 5.4-fold (*P*<0.001). The highest risk ratios of breast cancer death were observed among axillary lymph node positive patients where morphometrical grade III was associated with over 12-fold risk of breast cancer death as compared with morphometrical grade I. Instead, none of the traditional subjective grades produced statistically significant risk ratios of breast cancer death.

## CONCLUSIONS

In the medical literature, there is abundant confirmation on the prognostic significance of the histological grading of invasive breast cancer (Davis *et al*, 1986; Contesso *et al*, 1987, 1989; Elston and Ellis, 1991; Aaltomaa *et al*, 1991; Simpson and Page, 1994; Roberti, 1997; Page *et al*, 1998). Grading has also been acknowledged to have additional prognostic value in small (<1 cm in diameter) and axillary lymph node-negative tumours (Hopton *et al*, 1989; Stierer *et al*, 1992; Lee *et al*, 1996). When comparing the different methods to evaluate the prognosis of breast cancer, the predictive value of histological grading has been found to exceed that of hormone receptor status, DNA content, and c-erbB-2 or p5+3 expression (Fisher *et al*, 1988; Merkle *et al*, 1993; Macgrogan *et al*, 1995; Quenel *et al*, 1995). Although the bulk of information supports the prognostic correlations of breast cancer grading, other opinions also exist (Calle *et al*, 1986; Masters *et al*, 1987; Rosner and Lane, 1990; Johnson *et al*, 1992). The majority of criticism regarding breast cancer grading has centred on the lack of uniform methodology.

The morphometrical grading system is a method that applies quantitative morphometrical measurements and numerical assessment criteria for determining the degree of malignancy in invasive ductal breast cancer. In the present study we have examined the prognostic power of this modified grading system in predicting the outcome of 159 breast cancer patients. According to the current results, the morphometrical grading system very efficiently predicts the prognosis of breast cancer by dividing the patients into groups of favourable (grade I), intermediate (grade II) and unfavourable (grade III) outcome (log rank test *P*<0.0001). Moreover, patients were reliably classified into the different grades in prognostic subgroups divided on basis of axillary lymph node status and menopausal status. The prognostic significance of the morphometrical grading system is enhanced by the fact that the present analyses are based on follow-up information of a recent patient material representative of the era of mammographic screening and adjuvant treatment.

In this study we compared the prognostic value of the two grading methods according to the risk of breast cancer death associated with them. The risk of breast cancer death associated with the morphometrical grades in our material was 2.8 (*P*<0.005). In our material the morphometrical grade was especially efficient in determining patiens with the most unfavourable prognosis (grade III) (RR's varying between 1.2 to 5.1 in different prognostic subgroups). In multivariate analysis the risk ratios of the morphometrical grading system logically increased by each grade with grade III being associated with a 5.4-fold risk of breast cancer death (*P*<0.001).

In survival analysis the prognostic value of the subjective grading (Bloom and Richardson, 1957; Who, 1981) was clearly inferior to that of the morphometrical grading. The relatively short follow-up time can partly explain why the traditional subjective grades did not show prognostic significance in our material. The traditional subjective grades also differed decisively from the morphometrical grades. Application of the morphometrical grading led the classification towards the less malignant end of the scale as compared with the subjective grading. Moreover, the morphometrical grading system can be considered especially beneficial since it resulted in a diminished amount of cases representing an intermediate and consequently more uncertain outcome of disease (grade II).

Since breast cancer is known to be a heterogenous disease the nominal category of the diagnosis alone does not provide the clinician with sufficient information for treatment decisions. Therefore further classification systems, such as histological grading, have been developed. In light of the present results, the morphometrical grading system can be applied to clinical use as an aid in treatment decisions of patients with invasive ductal breast cancer. In addition to predicting the prognosis, the system could be used for standardisation and training of grading performance, and in the audit procedure of quality control.

The presented morphometrical grading system provides breast cancer grading new, more exact and reproducible principles, methods and criteria. In establishing new prognosticators, however, the clinical applicability of the method has to be considered. In a routine setting the morphometrical grading is obviously more laborious and time consuming than the traditional subjective grading. Morphometrical grading of one sample takes approximately 30 min which is considerably more than the time generally used in subjective grading. However, this does not necessarily increase the work load of the pathologist since the necessary morphometrical measurements can be performed by adequately trained laboratory personnel. The morphometrical grade can also be used in prognostication of certain patient subgroups, e.g. axillary lymph node positive patients. The morphometrical grading system could also be useful in standardising grading performance between laboratories, in training during pathology specialisation, and in clinical quality control. Also, the methods and criteria of the morphometrical grading system can be applied to the subjective grading of breast cancer in order to intensify the prognostic efficiency.

Development of a quantitative morphometrical grading system will require continued efforts. In the next phase it will be necessary to clarify the concomitant prognostic values of the grading features. This is especially motivated since previous studies suggest that the prognostic power of mitotic counts by far exceeds that of the other prognostic features (Clayton, 1991; van Diest and Baak, 1991). These findings could lead to development of a morphometric index where each grading feature would be provided with a coefficient reflecting the weight of the feature in prognostication. Application of artificial intelligence in the form of decision support systems would also be an elegant solution for unequal prognostic values (Ravdin and Clark, 1992; Ravdin *et al*, 1992). Finally, the results give a scientific medical basis for production of a future automated image analysis programme for morphometrical grading.
